# Do general practitioners adhere to the guideline on infectious conjunctivitis? Results of the Second Dutch National Survey of General Practice

**DOI:** 10.1186/1471-2296-8-54

**Published:** 2007-09-16

**Authors:** Remco P Rietveld, Gerben ter Riet, Patrick JE Bindels, François G Schellevis, Henk CPM van Weert

**Affiliations:** 1 Division of Clinical Methods & Public Health, Department of General Practice, Academic Medical Center, University of Amsterdam, Meibergdreef 15, 1105 AZ, Amsterdam; 2 Horten-Zentrum, Zürich, Switzerland; 3 Netherlands Institute for Health Services Research (NIVEL), Utrecht; 4 Department of General Practice/EMGO Institute, Vrije Universiteit Medical Center, Amsterdam

## Abstract

**Background:**

In 1996 the guideline 'The Red Eye' was first published by the Dutch College of General Practitioners. The extent to which general practitioners adhere to this guideline is unclear. Recently, data on the management of infectious conjunctivitis by general practitioners became available from the Second Dutch National Survey of General Practice. We measured the age-specific incidence of infectious conjunctivitis, described its management by Dutch general practitioners, and then compared these findings with the recommendations made in the guideline.

**Methods:**

In 2001, over a 12-month period, data from all patient contacts with 195 general practitioners were taken from electronic medical records. Registration was episode-oriented; all consultations dealing with the same health problem were grouped into disease episodes. Data concerning all episodes of infectious conjunctivitis (ICPC-code F70 and sub codes) were analysed.

**Results:**

Over one year, 5,213 new and recurrent episodes of infectious conjunctivitis were presented to general practitioners from a population of N = 375,899, resulting in an overall incidence rate of 13.9 per 1000 person-years, varying from more than 80/1000 py in children up to one-year old, to less than 12/1000 py in children over the age of 4. Topical ophthalmic ointments were prescribed in 87% of the episodes, of which 80% was antibiotic treatment. Fusidic acid gel was most frequently prescribed (69%). In most episodes general practitioners did not adhere to the guideline.

**Conclusion:**

In 2001, the management of infectious conjunctivitis by Dutch general practitioners was not in accordance with the recommendations of the consensus-based guideline published five years previously, despite its wide distribution. In 2006 this guideline was revised. Its successful implementation requires more than distribution alone. Probably the most effective way to achieve this is by following a model for systemic implementation.

## Background

The guideline entitled 'The Red Eye' was first published by the Dutch College of General Practitioners in 1996 [1]. The chapter concerning the management of infectious conjunctivitis recommends that, when infectious conjunctivitis is diagnosed, the general practitioner should emphasize to the patient that it is a contagious, but harmless disorder, which normally gets better on its own. Furthermore, the general practitioner should emphasize that if bacterial involvement is suggested by purulent secretion, antibiotic treatment might accelerate recovery. In that case, the general practitioner should ask the patient if they would prefer antibiotic treatment. In all other cases, regular cleaning of the infected eye with water is advised. Finally, the first and second choice antibiotics advised by the guideline are chloramphenicol and tetracycline. In 1996 there was no evidence from primary care-based trials on the treatment of infectious conjunctivitis. Hence, recommendations in the guideline were based upon consensus and secondary care-based trials [2]. Data on the management of infectious conjunctivitis in general practice from registration projects and studies, which took place before the guideline was published, were available [3-7]. These data showed that in more than 80% of cases of acute infectious conjunctivitis general practitioners prescribed ocular antibiotics. An interesting question is to what extent general practitioners have adhered to the guideline since its publication. The guidelines developed by the Dutch College of General Practitioners are normative and form an accepted base for daily clinical practice[2,8]. Furthermore, a large part of the curriculum of general practitioner trainees in the Netherlands is based upon these guidelines, as are continuing-education courses for general practitioners. Adherence to guidelines is reinforced when recommendations are more evidence-based, less controversial, and do not require changes in practice [9]. Recently, new data on the management of infectious conjunctivitis by general practitioners were made available by the Second Dutch National Survey of General Practice. This survey took place in 2001, five years after the introduction of the guideline [10]. It contains data from electronic medical records including diagnoses, prescribed medication and referrals to secondary care. Data from the survey provide insight into the management of infectious conjunctivitis in daily clinical practice. In this study we describe the age-specific incidence of clinically diagnosed infectious conjunctivitis and its management by general practitioners. These findings were then compared with the recommendations made in the guideline.

## Methods

### Dutch National Survey of General Practice

The study took place within the framework of the Second Dutch National Survey of General Practice, which was carried out in 2001 by the Netherlands Institute for Health Services Research (NIVEL) in collaboration with the National Information Network of General Practice (LINH). It included 61 general practices participating in the LINH network, and 43 additional general practices especially recruited for this survey. Thus, 104 general practices participated, with a total of 195 (164.75 FTE) general practitioners, and about 390,000 patients. This sample constitutes a representative group of Dutch primary care physicians (age, sex, geographical region, and urbanisation level of practice location), with a slight under-representation of single-handed general practitioners. All participating practices were computerised, had electronic patient files, and an electronic prescription system used for diagnosis-related prescribing. Data were extracted from all electronic medical records from each participating practice over a period of one year (mainly the calendar year 2001). Study design and methods of the survey have been published in more detail elsewhere[10].

### Infectious conjunctivitis

Data about all patient contacts included information on diagnosis, referrals, and prescriptions. Diagnoses were coded according to the International Classification of Primary Care (ICPC). A contact was defined as a face-to-face consultation of the patient with a general practitioner; contacts by telephone and contacts between patients and the medical receptionist were registered as a contact only if they led to a prescription or a referral. The registration was episode-oriented; all consultations dealing with the same health problem were grouped into disease episodes. The participating general practitioner decided whether a contact belonged to an existing disease episode or was the beginning of a new episode. A disease episode was coded by the diagnosis made at the most recent contact of that episode. Data on all episodes of infectious conjunctivitis (ICPC-code F70 and sub codes), age and sex of the patient, season, number of contacts with the general practitioner, prescriptions and referrals, were extracted.

### Analysis

The incidence of infectious conjunctivitis is represented as an incidence rate (per 1000 person-years). The numerator concerns new and recurrent episodes. A recurrent episode is a second (or even third or more) isolated episode of infectious conjunctivitis in the same patient within the one-year registration period. The denominator concerns the mid-time population size based upon the size and composition of the population at the start and at the end of the one-year registration period. The sex-specific incidence rate differences in age groups (overall, age 0–14, and 15 years and older) were calculated to investigate any difference between incidence rates in men and women in each age group. The season-specific incidence rate difference, spring and summer (from April to September) versus autumn and winter (from October to March), was also calculated.

The Poisson distribution was used to calculate the 95% confidence intervals of the incidence rates. Statistical analyses were performed using SPSS (version 11.5.2). We used Stata (version 9.1) to calculate the incidence rates and rate differences and their 95% confidence intervals.

## Results

### Incidence rates

In a one-year period, 5,213 new and recurrent episodes of infectious conjunctivitis were registered in a mid-time population of N = 375,899. The patients were representative of the general population in terms of age, sex and type of health care insurance (public/private). The incidence rate of infectious conjunctivitis was 13.9 episodes per 1000 person-years (95% CI: 12.5 to 15.3). The majority of the patients (96.1%) had one episode, 3.6% had two episodes, and 0.3% had three or more episodes of infectious conjunctivitis within one year. Table 1 shows the incidence rates of other conditions related to infectious conjunctivitis. The incidence rate indicates that, in a standard Dutch practice of 2350 patients, on average 32 (95% CI: 29 to 36) episodes of infectious conjunctivitis are recorded each year. This indicates that infectious conjunctivitis is ranked fifteenth in the list of most frequently seen diseases in primary care.

**Table 1:** Incidence rates of other conditions in relation to infectious conjunctivitis

ICPC-code	Condition	Incidence rate
F70	Infectious conjunctivitis	13,9
F72	Blepharitis, Hordeolum, and Chalazion	5,6
F71	Allergic conjunctivitis	3,3
F75	Foreign body	3,1
F02	Red eye	2,1
F03	Discharge	1,6
F73	Other infection eye	1,4
F85	Cornela ulcera	0,8

### Age, sex and seasonal relationships

**Figure 1:**
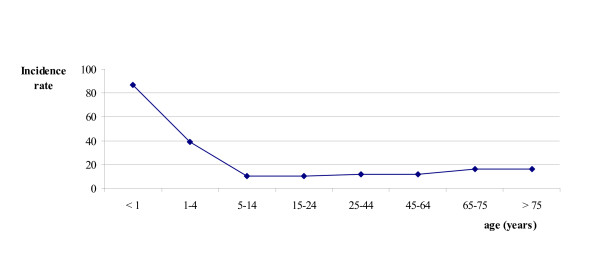
Age-specific incidence rates of infectious conjunctivitis (episodes per 1000 py).

Figure 1 shows that the incidence rate of infectious conjunctivitis in young children and patients aged over 65 was high compared to other age groups. Children up to 11 years of age accounted for almost 25% of all episodes. More episodes were recorded in women (14.9 per 1000 person-years) than in men (12.9 per 1000 person-years), incidence rate difference: 2.0 episodes per 1000 person-years (95% CI: 1.2 to 2.7). It seems that this difference is mostly caused by the difference in incidence of infectious conjunctivitis in adult women compared to men; the sex-specific incidence rate difference in the age group 15 years and older was 2.7 episodes per 1000 person-years (95% CI: 1.9 to 3.4). The sex-specific incidence rate difference in the age group 0 to 14 years was 0.4 episodes per 1000 person-years (95%CI: -1.8 to 2.6). We found no evidence of any seasonal influence on the incidence rate of infectious conjunctivitis (data not shown).

### Registration

Sixty-eight percent of the first contacts in an episode were registered as "infectious conjunctivitis", 26% as bacterial conjunctivitis and 6% as viral conjunctivitis. Most episodes (87%) consisted of one registered contact between patient and general practitioner.

### Management

**Figure 2:**
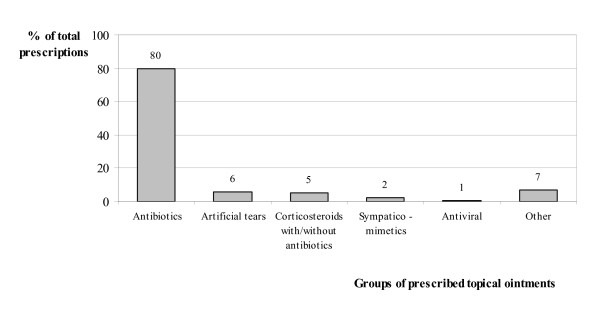
Distribution of prescriptions across different topical ophthalmic ointments (%).

**Figure 3:**
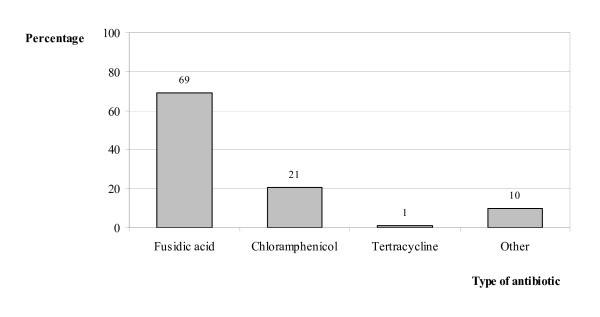
Choice of topical ophthalmic antibiotic; distribution across different antibiotics.

General practitioners prescribed a topical ophthalmic ointment to almost 87% of the patients with infectious conjunctivitis, 80% of which was antibiotic treatment (Figure 2). Fusidic acid gel was the most frequently prescribed of these (69%), followed by chloramphenicol (21%) (Figure 3). Surprisingly, 5% of all prescriptions consisted of ointments containing corticosteroids. (Figure 2).

Of all patients with one or more episodes of infectious conjunctivitis, 1.9% (n = 121) were referred to secondary care with a referral diagnosis 'infectious conjunctivitis'. Of these patients, 85% were referred to an ophthalmologist. There was no difference in referral rate per 1000 episodes in women compared to men, referral rate difference: 3.4 (95%CI: -5.1 to 11.8). Most referrals took place in the 25–44 year and 45–64 year age groups, 29% and 29% respectively.

### Adherence to the guideline

These results show that in most episodes of infectious conjunctivitis general practitioners do not adhere to the guideline. In 21% of episodes, general practitioners registered bacterial conjunctivitis in the electronic medical record. However, in more than two-thirds of all episodes general practitioners prescribed a topical antibiotic. Furthermore, fusidic acid was by far the most frequently prescribed antibiotic, which differs from the first choice antibiotic in the guideline.

## Conclusion

### Summary of main findings

The results show that, in 2001, five years after the publication of the guideline, the management of infectious conjunctivitis by general practitioners was predominantly not according to the guideline. Patients with infectious conjunctivitis received antibiotic treatment in almost 70% of episodes making the total number of prescriptions for antibiotics by general practitioners remarkably high. It is in contrast with the guideline's recommendations to limit the prescription of antibiotics to those patients with suspected bacterial involvement [1]. Although the guideline recommends chloramphenicol as the antibiotic of first choice, fusidic acid gel was by far most frequently prescribed. The data also show that almost all Dutch patients with an infectious conjunctivitis are managed in primary care.

The contrast between the guideline and daily practice may be explained as follows. The advice on treatment in the guideline was not based on randomised trials in patients typical of those seen in primary care, and therefore publications on this subject might not have caught the attention of general practitioners. Assumptions made in the guideline on the symptoms indicative of a diagnosis of infectious conjunctivitis, bacterial conjunctivitis in particular, were based on consensus. In a literature search we found no evidence to back up these assumptions [11]. Furthermore, consultations for conjunctivitis are often seen as a "catch up consultation" [12]. It is easier and less time consuming to give the patient the prescription they expect than to explain why a prescription is not necessary. Another reason is the preference or the expectation of the patient to be treated with an antibiotic. A British qualitative study, investigating why general practitioners prescribe antibiotics for infectious conjunctivitis in children, showed that the majority of parents of these children felt that treatment would help their child get better more quickly and most felt this treatment should be sought immediately. Furthermore, more than half of the parents felt that their child would not get better unless treated [12]. Finally, many day care centres and schools do not re-admit children before they have consulted their doctor, have been treated with antibiotics, or even until they are better. It should be noticed that this policy by day care centres and schools is in contradiction with recommendations made by Dutch municipal health services guidelines [13]. According to these guidelines, children with infectious conjunctivitis are not to be excluded from day care centres or school.

Surprisingly, 5% of all prescribed treatments contained corticosteroids, with or without antibiotics. It should be emphasised that this treatment for infective conjunctivitis is not recommended by our guidelines, and the general opinion is that corticosteroid prescriptions should only be initiated by ophthalmologists, after careful evaluation. The reasons for general practitioners to prescribe corticosteroids cannot be evaluated within our data. Possibly these prescriptions were repeat prescriptions first issued by ophthalmologists.

### Strengths and limitations of the study

The strength of this study is that the results can be extrapolated to the general Dutch population and Dutch general practitioners and probably also to other Western European countries. The population and general practices studied are representative of the Dutch situation. Furthermore, a Dutch study has shown that no differences exist in working style between those general practitioners who participated in a registration network and those who did not. This also holds for general practitioners working in computerised or non-computerised practices [14]. However, it should be noted that the survey contained a relatively low number of single-handed general practitioners. A limitation of this study might be the fact that the data extraction was focused on ICPC-code F70 and sub codes only, as coded on the diagnostic line in the Electronic Medical Record. This means that our findings depend strongly on the quality of registration by the general practitioners. However, participating general practitioners were trained and experienced users of electronic medical records and ICPC. They did not differ from other general practitioners in diagnosing disease episodes [15]. Inter-doctor variation in ICPC coding of participating general practitioners was small and the level of agreement with external ICPC experts (n = 4) was on average 81% [15].

Another limitation might be the fact that only face-to-face consultations were registered. Contacts by telephone and with the receptionist that did not lead to a well-defined action were not registered. For example, a question from a patient about a red eye which did not lead to any action by the general practitioner or receptionist would have been missed. Therefore, there might be some under-representation of cases of infectious conjunctivitis in which no treatment was given. However, this proportion of patients will be small. First, only 15% of all contacts were telephone contacts [16]. Second, most telephone contacts about infectious conjunctivitis will either lead to a prescription or advice.

### Comparison with existing literature

Over two-thirds of the episodes of infectious conjunctivitis were treated with topical antibiotics, almost seventy percent of those with fusidic acid. Although not comparable to the Second Dutch National Survey of General Practice, an English study performed in January 2001 indicates that the total number of prescriptions by English general practitioners might even be higher. In this postal questionnaire survey on the management of acute infectious conjunctivitis among of 303 general practitioners in Southern England, 95% of the 234 responding general practitioners indicated they usually prescribed topical antibiotics for acute infectious conjunctivitis [17]. In fact, in England, 3.4 million community prescriptions for topical ocular antibiotics are issued each year, and in the Netherlands more than 900,000 prescriptions for topical ocular antibiotics were issued in 2001. Primary care physicians issued 85% of these prescriptions[18,19].

In 2003 in the United Kingdom, chloramphenicol and fusidic acid gel were the first and the second most prescribed topical ocular antibiotics. About 25% of all topical ocular antibiotic prescriptions were for fusidic acid [19]. This is in line with the results from the survey by Everitt et al., in which 13% (95%CI: 8–17) of the prescriptions concerned fusidic acid gel, compared to 87% (95%CI: 83–92) prescriptions of chloramphenicol [17].

The prescription policy of Dutch general practitioners may be explained by the fact that fusidic acid gel needs to be administered less frequently than chloramphenicol eye drops, and also, it appears to have no serious adverse effects [20].

After the guideline appeared, the results of two trials, conducted in primary care were published, [21,22]. These results backed up the recommendations on treatment made in the guideline. In one of these trials we had compared fusidic acid gel 1% to placebo in adult patients with acute infectious conjunctivitis. We found that after seven days, recovery rates in the fusidic acid gel and placebo groups were essentially the same. Furthermore, 65% of the 58 isolates found in 181 patients were resistant to fusidic acid. The only pathogen not resistant to fusidic acid was *Staphylococcus aureus*, accounting for 13 of the 58 isolates [21]. Rose et al. studied the effect of chloramphenicol treatment for acute infectious conjunctivitis in children aged 6 months to 12 years compared to placebo [22]. Their findings were essentially the same; most children will get better by themselves equally quickly and thus do not need antibiotic treatment.

### Implications for clinical practice

The question is whether general practitioners should prescribe antibiotics at all to patients with infectious conjunctivitis. The prevalence of bacterial origin in our trial was 32% [21]. In a similar population Everitt et al. found a bacterial pathogen rate of 50%, whereas Rose et al. found a bacterial pathogen rate of 78% in children [22,23]. As these studies show, the spontaneous resolution rate of bacterial conjunctivitis is high. However, we also found that in culture-positive patients the treatment effect tended to be strong [21]. Furthermore, a diagnostic study by our group showed that it might be possible for the general practitioner to identify bacterial origin of infectious conjunctivitis by asking the patient three questions, namely about gluey eyes in the morning, itching, and a history of infectious conjunctivitis [24]. Asking these questions is a good way of identifying those patients who have a bacterial infection and those who do not. However, this diagnostic rule has not been validated yet. Therefore, at this time, we must accept that it is difficult to differentiate clinically between bacterial and viral conjunctivitis. As shown by Everitt et al., no or a delayed prescription is a safe strategy [23]. Following this policy will help to reduce unnecessary prescription of antibiotics.

In 2006 the 'The Red Eye' guideline was revisited [25]. An major part of the revision was based on the primary care trials already referred to in this discussion [21,22]. Its successful implementation requires more than distribution alone. Probably the most effective way to achieve this is by following a model for systemic implementation [26].

In conclusion, over two-thirds of episodes of infectious conjunctivitis are treated with topical antibiotics, almost seventy percent of these with fusidic acid. This is at odds with the recommendations of the Red Eye guideline and recently published evidence on this subject. If the patient specifically wants antibiotics then they should only be prescribed if there is a high probability of a bacterial origin.

## Competing interests

The author(s) declare that they have no competing interests.

## Authors' contributions

RPR and GtR analyzed the data and RPR wrote the first draft of the manuscript. All other authors gave comments on the first to the last versions of the manuscript. All authors read and approved the final manuscript.

## Pre-publication history

The pre-publication history for this paper can be accessed here:


